# Development of an in chemico high-throughput screening method for the identification of skin sensitization potential

**DOI:** 10.1007/s00204-023-03550-z

**Published:** 2023-07-19

**Authors:** Isabel Ferreira, Gonçalo Brites, Ana Silva, Francisco Caramelo, Bárbara Oliveiros, Bruno Miguel Neves, Maria Teresa Cruz

**Affiliations:** 1 Center for Neuroscience and Cell Biology (CNC), University of Coimbra, Coimbra, Portugal; 2 Faculty of Pharmacy, University of Coimbra, Coimbra, Portugal; 3 Toxfinder LDA, IPN - Instituto Pedro Nunes, Coimbra, Portugal; 4 Laboratory of Biostatistics and Medical Informatics, Faculty of Medicine, University of Coimbra, Coimbra, Portugal; 5 Center for Innovative Biomedicine and Biotechnology (CIBB), University of Coimbra, Coimbra, Portugal; 6 Department of Medical Sciences and Institute of Biomedicine – iBiMED, University of Aveiro, Aveiro, Portugal

**Keywords:** Skin sensitization, Direct peptide reactivity assay, In chemico method, Low-molecular-weight chemicals

## Abstract

It is well established that chemical-peptide conjugation represents the molecular initiating event (MIE) in skin sensitization. This MIE has been successfully exploited in the development of *in chemico* peptide reactivity assays, with the Direct Peptide Reactivity Assay (DPRA) being validated as a screening tool for skin sensitization hazard as well as an OECD test guideline. This test relies on the use of a high-performance liquid chromatography/ultraviolet detection method to quantify chemical-peptide conjugation through measurement of the depletion of two synthetic peptides containing lysine or cysteine residues, which is labor-intensive and time-consuming. To improve assay throughput, sensitivity, and accuracy, we have developed a spectrophotometric assay for skin sensitization potential based on MIE measurement—the ProtReact assay. ProtReact is also a cheaper, faster, simpler, and more accessible alternative for the DPRA, giving comparable results. A set of 106 chemicals was tested with ProtReact and the peptide depletion values compared with those reported for the DPRA. The predictive capacity of both assays was evaluated with human reference data. ProtReact and DPRA assays show similar predictive capacities for hazard identification (75% and 74%, respectively), although ProtReact showed a higher specificity (86% versus 74%, respectively) and lower sensitivity (69% versus 73%). Overall, the results show that ProtReact assay described here represents an efficient, economic, and accurate assay for the prediction of skin sensitization potential of chemical haptens.

**Supplementary Information:**

The online version contains supplementary material available at 10.1007/s00204-023-03550-z.

## Introduction

Allergic contact dermatitis (ACD) is a delayed hypersensitivity reaction that affects about 20% of the European population (Uter et al. 2020). The identification of potential hazard has relied heavily on in vivo methods, although extensive research efforts have been placed on the development of new approach methodologies (NAMs). Indeed, animal testing in the European Union is already prohibited since 2004 for cosmetic products (“testing ban”). In 2009, the European Union also prohibited the marketing of cosmetic products containing ingredients which have been tested on animals (“marketing ban”), (European Commission 2013). For the most complex human health effects (e.g., skin sensitization), the marketing ban was postponed until 11 March 2013, when a full ban on animal testing entered into force, irrespective of the availability of alternative non-animal tests (Taylor and Rego Alvarez 2020, European Commission 2013). Consequently, the development of NAM *for* skin sensitization has been encouraged, and considerable efforts have been made (OECD TG 442C, 442D, 442E, and 497). The available NAM was developed based on the Adverse Outcome Pathway (AOP) of skin sensitization, which provides the mechanistic basis for the integration of skin sensitization-related information (OECD 2014). The key events in the AOP include covalent binding of a chemical to skin proteins, keratinocytes’ activation, dendritic cells’ activation, and T cells’ activation and proliferation, which ultimately leads to skin sensitization (OECD 2014). Several tests have already been validated for hazard assessment and have OECD Test Guidelines in place. Indeed, Direct Peptide Reactivity Assay (DPRA), developed by Gerberick and coworkers (Gerberick et al. 2004, 2007), was one of the first *in chemico* methods to be validated as a screening tool for skin sensitization hazard as well as an OECD test guideline (OECD TG 442C), also being part of several defined approaches for skin sensitization (Kleinstreuer et al. 2018). DPRA addresses the reactivity of the test chemical with either synthetic peptides containing Cysteine (Ac-RFAACA-COOH) or Lysine (Ac-RFAAKAA-COOH) following 24 h of incubation. Cysteine and lysine percent peptide depletion is then determined using HPLC–UV method, and a threshold of 6.38% mean Cys- and Lys-peptide depletion is used to discriminate between skin sensitizers and non-sensitizers. Some of the limitations of the current DPRA include the requirement of high concentration peptides and chemicals that often interfere with the UV detection method, leading to false-positive or false-negative results. Furthermore, HPLC method is labor-intensive and time-consuming, and HPLC equipment is not readily available in many laboratories, always requiring expert personnel.

Herein, we describe the implementation of a peptide competitive assay, using a spectrophotometric approach, which may improve throughput, screening efficiency, and cost-effectiveness. The ProtReact detection assay is based on matrices functionalized with peptides, in which test chemical haptenization is determined by the spectrophotometric color change of specific reactive probes. This study compared the performance of the ProtReact detection assay with the DPRA.

## Materials and methods

### Materials

The chemicals poly-l-lysine hydrobromide, l-glutathione reduced, DMSO, as well as chemical sensitizers and non-sensitizers were obtained from Sigma-Aldrich Chemical Co. (St. Louis, MO, USA). Some of the chemical sensitizers and non-sensitizers were also provided by Cosmetics Europe. The CAS registry numbers of tested chemicals are given in Table 1. The two probes tested in this assay, Ellman's reagent [5,5′-dithiobis-(2-nitrobenzoic acid) or DTNB] and Fluorescein-5-EX, Succinimidyl Ester (FSE) were from Sigma-Aldrich Chemical Co and Invitrogen (Invitrogen, USA), respectively. The thiol functional agarose beads (AR-TH-1) were from NANOCS (Nanocs Inc., NY, USA). The black 96-well glass-bottom microplate plates (#4580) used were from Corning (Corning Glass Works, Corning, NY). Colorimetric and fluorescent readouts were detected using a Synergy™ HT BioTek plate reader (BioTek Instruments, Inc., Winooski, VT, USA).

**Table 1:** Depletion of cysteine and lysine peptide measured by ProtReact and DPRA

	Chemical name	CAS	Human potency category^2^	Cys depletion (%)		Lys depletion (%)		Mean depletion (%)	
				ProtReact	DPRA^1^	ProtReact	DPRA^1^	ProtReact	DPRA^1^
**1**	2,4-Dinitrochlorobenzen	97-00-7	1	62.0	100	53.6	14.7	57.8	57.4
2	Methylisothiazolinone	2682-20-4	1	10.0	97.9	0.0	0	5.0	49.0
3	1,4-Phenylenediamine	106-50-3	1	22.3	93.0	32.3	23.5	27.3	58.3
4	Tetrachlorosalicylanilide	1154-59-2	1	51.0	36.8	80.8	9.0	65.9	22.9
5	Dimethyl fumarate	624-49-7	1	97	100	8.0	42.9	52.5	71.5
6	Diphencyclopropenone	886-38-4	1	6.3	98.8	29.8	0.0	18.0	49.4
7	Benzisothiazolinone	2634-33-5	2	86.0	97.7	25.3	9.7	55.6	53.7
8	Cinnamic aldehyde	104-55-2	2	54.5	70.6	32.0	43.3	43.3	57.0
9	Diethyl maleate	141-05-9	2	67.5	100.0	16.8	85.5	42.1	92.8
10	3-Dimethylaminopropylamine	109-55-7	2	0.0	10.2	0.0	0.0	0.0	5.1
11	Formaldehyde	50-00-0	2	13.7	60.4	40.0	11.2	26.8	35.8
12	Glutaraldehyde	111-30-8	2	43.3	30.2	63.0	85.4	53.1	57.8
13	Glyoxal	107-22-2	2	24.0	92.6	42.0	88.9	33.0	90.8
14	Lyral	31906-04-4	2	39.4	39.4	0.0	3.4	19.7	21.4
15	Isoeugenol	97-54-1	2	100.0	89.3	23.0	10.7	61.5	50.0
16	Lauryl gallate	1166-52-5	2	93.8	90.9	83.3	8.7	88.5	49.8
17	Methyl heptine carbonate	111-12-6	2	39.3	97.2	0.0	0.0	19.7	48.6
18	Methyldibromo glutaronitrile*	35691-65-7	2	81.2	100.0	47.0	28.6	64.1	64.3
19	2-Nitro-1,4-phenylenediamine*	5307-14-2	2	49.6	93.3	42.8	0.0	46.2	46.7
20	Propyl gallate	121-79-9	2	20.8	59.9	44.0	26.6	32.4	43.3
21	Thioglycerol*	96-27-5	2	0.0	27.3	0.0	28.4	0.0	27.9
22	Toluene diamine sulfate	615-50-9	2	72.0	78.4	96.0	15.0	84.0	46.7
23	Methyl 2-nonynoate	111-80-8	2	100	100	4.9	3.2	52.4	51.6
24	2-Methoxy-4-methylphenol	93-51-6	2	9.5	0	5.6	11.5	7.6	5.8
25	Trans-2-hexenal	6728-26-3	2	86	97.9	25.0	3.6	55.5	50.8
26	2-Aminophenol	95-55-6	2	35.7	96.2	40.0	18.1	37.9	57.2
27	Abietic acid	514-10-3	3	63.3	99.9	29.5	16.3	46.4	58.1
28	Benzoyl peroxide	94-36-0	3	92.7	100.0	87.7	81.3	90.2	90.7
29	Bisphenol A diglycidyl ether	1675-54-3	3	48.0	39.4	8.7	3.4	28.4	21.4
30	Butyl glycidyl ether	2426-08-6	3	43.0	67.3	0.0	11.5	21.5	39.4
31	Chlorpromazine	50-53-3	3	39.5	− 19.6	21.0	0.0	30.3	− 9.8
32	Cinnamic alcohol	104-54-1	3	14.2	0.0	0.0	15.1	7.1	7.6
33	Citral	5392-40-5	3	29.0	85.7	52.7	16.9	40.8	51.3
34	Coumarin	91-64-5	3	4.7	1.0	0.0	0.0	2.3	0.5
35	Ethylene diamine	107-15-3	3	3.0	3.4	0.0	0.0	1.5	1.7
36	Eugenol	97-53-0	3	21.8	9.2	2.0	19.2	11.9	14.2
37	Farnesol	4602-84-0	3	37.0	7.3	24.0	0.0	30.5	3.7
38	Glyceryl monothioglycolate*	30618-84-9	3	0.0	0.6	0.0	18.6	0.0	9.6
39	1,4-Dihydroquinone	123-31-9	3	77.3	83.3	37.0	51.1	57.2	67.2
40	Imidazolidinyl urea	39236-46-9	3	16.0	52.3	52.8	1.3	34.4	26.8
41	2-Mercaptobenzothiazole	149-30-4	3	8.3	97.5	17.0	0.0	12.6	48.8
42	5-Methyl-2,3-hexanedione	13706-86-0	3	10.5	25.8	0.0	7.5	5.3	16.7
43	Metol	55-55-0	3	60.0	100	48.3	44.7	54.1	72.4
44	Penicillin G	61-33-6	3	1.5	14.3	0.0	0.0	0.8	7.2
45	Phenyl benzoate	93-99-2	3	17.0	36.8	0.0	19.6	8.5	28.2
46	Tetramethylthiuram disulfide	137-26-8	3	100.0	99.5	6.0	6.9	53.0	53.2
47	3-Propylidenephthalide	17369-59-4	3	86.0	14.30	82.0	30.60	84.0	22.5
48	Allyl phenoxyacetate	7493-74-5	3	12.6	0.6	0.0	4.1	6.3	2.3
49	Cinnamyl nitrile	1885-38-7	3	16.6	4	10.9	0.0	13.7	2.0
50	Phenylacetaldehyde	122-78-1	3	100	60.7	32.0	22.6	66.0	41.7
51	Dibenzyl ether	103-50-4	4	95.8	11.38	0.0	0.00	47.9	5.7
52	Benzyl alcohol	100-51-6	4	0.5	0.00	0.0	0.00	0.3	0.0
53	Amyl cinnamic aldehyde	122-40-7	4	24.0	0.6	0.0	3.9	12.0	2.2
54	Amylcinnamyl alcohol	101-85-9	4	8.8	5.0	5.3	0.0	7.0	2.5
55	Aniline	62-53-3	4	3.3	0.0	3.0	9.7	3.1	4.9
56	Benzocaine	94-09-7	4	2.3	29.2	2.0	0.0	2.1	14.6
57	Carvone	6485-40-1	4	19.3	25.1	0.0	0.6	9.6	12.9
58	Ethyl acrylate	140-88-5	4	82.8	96.4	7.0	93.7	44.9	95.1
59	Ethyleneglycol dimethacrylate	97-90-5	4	22.6	87.3	15.5	12.4	19.1	49.8
60	Geraniol	106-24-1	4	0.8	0.0	0.0	10.0	0.4	5.0
61	Hexyl salicylate	6259-76-3	4	2.0	3.9	0.0	1.4	1.0	2.7
62	Hydroxycitronellal	107-75-5	4	5.0	17.5	3.8	6.5	4.4	12.0
63	Iodopropynyl butyl carbamate	55406-53-6	4	85.3	99.7	3.3	0.0	44.3	49.9
64	Lilial	80-54-6	4	52.0	14.0	17.0	0.7	34.5	7.4
65	Linalool	78-70-6	4	22.3	0.0	0.0	7.9	11.1	4.0
66	Methylmethacrylate	80-62-6	4	4.8	36.7	0.0	10.0	2.4	23.4
67	Resorcinol	108-46-3	4	1.0	1.6	0.0	0.0	0.5	0.8
68	α-Methyl cinnamic aldehyde	101-39-3	4	20.8	10.4	5.1	28.8	13.0	19.6
69	Β, β 3-trimethyl benzenepropanol	103694-68-4	4	22.6	0.1	0.0	1.4	11.3	0.8
70	Benzyl cinnamate	103-41-3	4	15.7	2.2	0.0	3.2	7.9	2.7
71	Isocyclocitral	1335-66-6	4	30.3	15.9	0.0	42.4	15.2	29.1
72	Anethole	104-46-1	5	40.8	0.0	0.0	9.6	20.4	4.8
73	Anisyl alcohol	105-13-5	5	0.0	35.5	3.5	100.0	1.8	67.8
74	Benzyl benzoate	120-51-4	5	6.0	0.2	0.0	3.0	3.0	1.6
75	Benzyl salicylate	118-58-1	5	4.8	3.8	3.0	1.5	3.9	2.7
76	Citronellol	106-22-9	5	17.3	14.4	0.0	0.0	8.6	7.2
77	Hexyl cinnamic aldehyde	101-86-0	5	0.0	0.0	18.0	0.0	9.0	0.0
78	Isopropyl myristate	110-27-0	5	0.0	0.8	0.0	0.0	0.0	0.4
79	Limonene	5989-27-5	5	43.8	4.9	0.0	1.3	21.9	3.1
80	Pentachlorophenol	87-86-5	5	0.0	0.0	35.0	14.5	17.5	7.3
81	Propyl paraben	94-13-3	5	1.2	8.2	0.0	0.0	0.6	4.1
82	Pyridine	110-86-1	5	0.0	1.5	0.0	0.0	0.0	0.8
83	Triethanolamine	102-71-6	5	0.0	0.0	6.0	3.1	3.0	1.6
84	Diethanolamine	111-42-2	5	0.3	5.9	0.0	2.2	0.1	4.1
85	Hydrocortisone	50-23-7	5	3.0	39.1	1.4	82.9	2.2	61.0
86	Isopropanol	67-63-0	5	1.4	0.0	6.5	0.5	4.0	0.3
87	Propylene glycol	57-55-6	5	1.0	0.0	0.0	0.6	0.5	0.3
88	α-Methyl-1,3-benzodioxole- 5-propionaldehyde	1205-17-0	5	50.6	44.9	28.4	3.7	39.5	24.3
89	Vanillin	121-33-5	5	9.3	3.2	4.0	0.0	6.7	1.6
90	Methyl salicylate	119-36-8	5	11	0.3	6.0	1.6	8.5	1.0
91	Benzaldehyde	100-52-7	5	9.3	7.2	0.0	0.0	4.7	3.6
92	Phenoxyethanol	122-99-6	5	12.3	11.4	4.0	25.4	8.2	18.4
93	4-Aminobenzoic acid	150-13-0	5	9.7	10.7	0.0	0.4	4.9	5.6
94	1-Butanol*	71-36-3	6	0.0	0.0	0.0	1.2	0.0	0.6
95	Diethyl phthalate	84-66-2	6	4.7	0.8	0.0	0.0	2.3	0.4
96	Dimethylsulfoxide	67-68-5	6	0.3	0.4	0.0	1.2	0.1	0.8
97	Glycerol	56-81-5	6	5.0	0.0	14.0	2.1	9.5	1.0
98	Hexane	110-54-3	6	5.3	0.0	0.0	0.0	2.7	0.0
99	Lactic acid	50-21-5	6	0.2	0.0	0.0	0.8	0.1	0.4
100	Salicylic acid	69-72-7	6	0.8	3.5	5.0	21.1	2.9	12.3
101	Sodium lauryl sulfate	151-21-3	6	0.0	0.0	16.3	0.0	8.1	0.0
102	Tocopherol*	59-02-9	6	0.0	0.0	85.0	7.1	42.5	3.6
103	Xylene	1330-20-7	6	0.8	9.7	0.0	0.0	0.4	4.9
104	Phenol	108-95-2	6	13.7	15.4	9.7	0.0	11.7	7.7
105	Tween 80	9005-65-6	6	18.5	49.1	0.0	12.9	9.3	31.0
106	Octanoic acid	124-07-2	6	13	0	22.7	0.9	17.8	0.5

Data expressed as mean of at least three independent experiments

^1^Hoffmann et al. (2018)

^2^Basketter et al. (2014)

*Chemicals that interfered with GSH in solution assay and/or were colored chemicals. Cysteine depletion was measured with thiol-functionalized agarose beads

### Test substances and evaluation of available data

For the evaluation of ProtReact and DPRA predictive capacity, previously published standard DPRA data were retrieved from Hoffman and colleagues (Hoffmann et al. 2018) which curated a comprehensive database of 128 substances with multiple outputs [e.g., DPRA data, lymph-node assay (LLNA) data, human data, among others]. Chemicals were identified as sensitizers or non-sensitizers according to human data published by Basketter and colleagues, which identified 6 categories of human sensitizing potency, with 1 being the most potent and 5 the least potent; category 6 represented true non-sensitizers (Basketter et al. 2014). Given that category 5 contains substances that have a very low intrinsic ability to cause skin sensitization and sensitization in the general population is likely to be extremely rare (Basketter et al. 2014), for the purpose of this article, the chemicals in category 5 and 6 were classified as non-sensitizers.

### Preparation of controls and test chemicals

Test chemicals (TCs) and control substances were prepared on the day of testing, immediately before use. The test chemicals were pre-weighted into 1.5 mL test tubes and dissolved in 1 mL of DMSO to prepare a 100 mM solution (the weight of the test chemical to be added to the vial was determined based on the molecular weight and purity). This stock solution was further diluted in each assay to a working concentration of 5 mM.

**Fig. 1:**
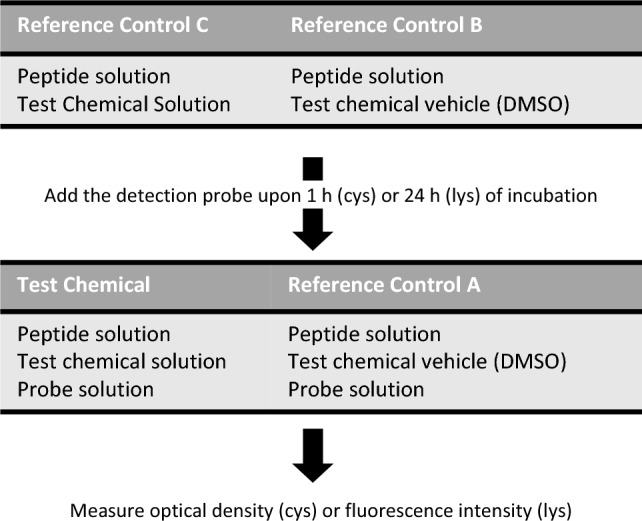
Schematic representation of the ProtReact assay. Cys, cysteine; Lys, lysine

To guarantee the performance of the assay, several controls were performed. 1-Fluoro-2,4-dinitrobenzene (DNFB) was used as positive control and lactic acid (LA) as negative control. Three reference controls were also included: (1) Reference control A, used to calculate the maximum fluorescence or absorbance in the presence of lysine or cysteine-containing peptides, consisted of the incubation of peptides with chemical’s solvent (DMSO) and probes (FSE or Ellman’s reagent); (2) Reference control B, which represents the blank assay and consisted in the incubation of peptides with DMSO, without incubation with probes; and (3) Reference control C, used to verify whether the test chemical or positive and negative controls interferes with the detection method, which consists in the incubation of peptides with test chemicals, without incubation with probes (Fig. 1). All controls were included in each run.

### ProtReact assay design

The ProtReact assay measures the reactivity of the test chemical in: (1) 96-well plates functionalized with lysine groups during 24 h and (2) glutathione in solution during 1 h of incubation as described in Supplementary Figs. S1 and S2. Following incubation with the test chemicals, and based on a competitive-binding assay, the 96-well plates or glutathione in solution is further exposed to a lysine or thiol-reactive fluorogenic or chromogenic reagent, respectively. The readout is the measurement of fluorescence/absorbance in a microplate reader, which is widely used in many laboratories. High and low fluorescence/absorbance indicates low and high reactivity, respectively. Cysteine depletion and lysine depletion assays were performed concomitantly, but can also be performed on separate days. At least three individual runs/experiments were performed for each assay.

### Cysteine depletion

Cysteine depletion was assessed with glutathione in solution, although, and since colored chemicals interfere with the detection method, an alternative assay with thiol agarose beads was developed for colored chemicals. Briefly, test chemicals were first dissolved in DMSO to prepare a 100 mM solution and a working solution of 5 mM was used in each assay. For cysteine depletion in solution, 10 µL of 100 mM test chemical solution was incubated with 190 µL of 0.2 mM GSH solution in 0.1 Phosphate Buffer (PB), pH 8 in a test tube. The mix was allowed to react for 1 h at room temperature (RT), in a test tube rotator protected from light. After 1 h of incubation, 20 µL of a 10 mM solution of the thiol-sensitive colorimetric reagent Ellman’s reagent was added and allowed to incubate for 10 min. The amount of colorimetric product formed was then quantified at 405 nm on Synergy HT plate reader (BioTek Instruments, Winooski, VT, USA). For colored chemicals, 10 µL of 100 mM test chemical solution, 140 µL of 30% (v/v) ethanol, and 50 µL of thiol function agarose beads were incubated for 1 h at RT in a test tube rotator. Beads were then centrifuged at 9600*g* for 1 min and washed with 100 µL of PB, pH 8 to remove the unbound test chemicals. 130 µL of PB and 20 µL of 10 mM Ellman’s reagent (thiol-sensitive colorimetric reagent) were then added to the beads and incubated in the test tube for 10 min. The amount of colorimetric product formed was then quantified at 405 nm on Synergy HT plate reader.

### Lysine depletion

For lysine depletion, a black 96-well bottom glass plate (Corning ref 4580) was first coated with 0.01% Poly-l-Lysine Hydrobromide (PLL; 50 µL/well) for 2 h at 37 °C or 24 h at RT. The wells were then washed three times with phosphate-buffered saline and 70 µL of 5 mM solution of the test chemicals or controls were added to each well (two replicates were performed for each chemical) and allowed to incubate for 24 h at 37 °C. Wells were then washed again with PBS and incubated with 70 µL of 10 µM FSE, a lysine-reactive fluorogenic reagent, for 1 h in the dark at 37 °C. Fluorescence intensity was then detected at Exc 485 ± 20 nm Em 528 ± 20 nm on a microplate reader.

### Evaluation of probe depletion

The dataset consisted of 106 chemicals, of which 71 were sensitizers and the remaining 35 were non-sensitizers, according to human data (Table 1). The putative reaction mechanism for each chemical was retrieved from Hoffman and colleagues (Hoffmann et al. 2018) and is provided in Supplementary Table S1. Depletion of the probe molecule indicates whether a substance is a binder or non-binder. Therefore, the cysteine and lysine percent peptide depletion was calculated for absorbance and fluorescence measurements, respectively, using the following equations for negative control (NC), positive control (PC) or test chemical (TC)﻿:

1 $$\mathrm{NC}\%\; \mathrm{Peptide \;Depletion}=\left[1-\left(\frac{{\text{NC}}-{\text{C}}}{{\text{A}}-{\text{B}}}\right)\right]\times 100$$

2 $$\mathrm{PC}\% \; \mathrm{Peptide \; Depletion}=\left[1-\left(\frac{{\text{PC}}-{\text{C}}}{{\text{A}}-{\text{B}}}\right)\right]\times 100$$

3 $$\mathrm{TC}\% \; \mathrm{Peptide\; Depletion}=\left[1-\left(\frac{{\text{TC}}-{\text{C}}}{{\text{A}}-{\text{B}}}\right)\right]\times 100$$

NC—negative control, PC—positive control, TC—test chemical, A—reference control A, B—reference control B, and C—reference control C.

### Prediction model

The mean value of the cysteine and lysine percent peptide depletion was calculated for reference controls, and positive and negative controls, and for each test chemical. Upon calculating the mean, negative depletion values were considered as 0% and depletion values above the maximum depletion considered as 100%. For discrimination of sensitizers and non-sensitizers in the ProtReact assay, a cut-off value was calculated using an ROC analysis: chemicals were classified as sensitizers if the cysteine percent peptide depletion values were equal or higher than 13.935% or the mean of cysteine and lysine percent peptide depletion values were equal or higher than 9.563%. For DPRA, chemicals were classified as sensitizers if the mean of cysteine and lysine percent peptide depletion was higher than 6.38% (Table 2, Supplementary Table S1). If these criteria were not met, the chemical was classified as a non-sensitizer.

**Table 2:** ProtReact and DPRA prediction models

	ProtReact	DPRA	Prediction
Cysteine % depletion	≥ 13.935%	> 13.89%^a^	Sensitizer
Mean of cysteine and lysine % depletion	≥ 9.563%	> 6.38%	

^a^Cysteine depletion is used when co-elution of the test chemical occurs only with the lysine peptide

## Results

### Cysteine and lysine depletion

A dataset of 106 chemicals was tested for cysteine and lysine depletion using the ProtReact assay and the results obtained are reported in Table 1. The chemicals Methyldibromo glutaronitrile (T18), 2-Nitro-1,4-phenylenediamine (T19), Thioglycerol (T21), Glyceryl monothioglycolate (T38), 1-Butanol (T94), and Tocopherol (T102) interfered with GSH in solution approach and/or were colored chemicals, and therefore, cysteine depletion was assayed using the thiol agarose beads’ approach. Overall, the results for the conventional DPRA assay and ProtReact showed to be quite similar for the 106 chemicals. Although, DPRA showed higher cysteine depletion values and ProtReact higher values for lysine depletion (Table 1 and Supplementary Figs. S3 and S4).

### Human hazard prediction

In the current study, we also compared the hazard prediction achieved with each assay for the 106 chemicals. According to DPRA (OECD TG 442C), chemicals can be classified as sensitizers if the mean of cysteine and lysine % depletion is higher than 6.38%. In cases where co-elution occurs with lysine peptide, chemicals are classified as sensitizers when cysteine % depletion is higher than 13.89%. For ProtReact, chemicals were classified as sensitizers if the cysteine % depletion values were equal or higher than 13.935% or the mean of cysteine and lysine % depletion values were equal or higher than 9.563%. If these criteria were not met, the chemical was classified as a non-sensitizer (NS) (Table 2, Supplementary Table S1). Chemical hazard classification was then compared with the human data available (reviewed in Basketter et al. 2014). Chemicals classified as human categories 1–4 were considered sensitizers (S) and as categories 5–6 were considered non-sensitizers (Basketter et al. 2014).

The predictive parameters for hazard prediction showed to be in a comparable range for the two approaches, with ProtReact approach (cysteine depletion) showing the highest accuracy and balanced accuracy and the highest specificity, although with a lower sensitivity (Table 3). DPRA prediction model is based on mean percent cysteine and percent lysine depletion, only considering the cysteine depletion when co-elution occurs only with the lysine peptide. Since ProtReact showed a slightly better performance with cysteine depletion only, detailed hazard prediction comparisons were performed contemplating only cysteine depletion for ProtReact and mean-peptide depletion for DPRA (Table 4).

**Table 3:** Performance measurements for ProtReact and DPRA

Chemical name	ProtReact		DPRA	
	Cys depletion > 13.935%	Mean depletion > 9.563%	Cys depletion > 13.89%	Mean depletion > 6.38%
Accuracy	**75%**	73%	73%	74%
Balanced accuracy	**77%**	75%	75%	74%
Sensitivity	69%	69%	68%	**73%**
Specificity	**86%**	80%	83%	74%

Accuracy = (TP + TN)/(TP + FP + FN + TN); balanced accuracy = (sensitivity + specificity)/2; sensitivity = TP/(TP + FN)

Specificity = TN/(TN + FP); Cys—cysteine, Lys—lysine

The highest performance measurements are highlighted in bold

**Table 4:** Hazard classification with DPRA and ProtReact depletion data: an overview

	ProtReact	DPRA	Concordance	Chemicals (table number)^#^	Mechanistic domain
Hazard classification	–	–	79		
Correctly classified	79	78	65		
False positives	5	9	3	**Common**	
				Citronellol (76)	None
				α-Methyl-1,3-benzodioxole- 5-propionaldehyde (88)	Schiff base
				Tween 80 (105)	None
				**ProtReact**	
				Anethole (72)	Michael acceptor^§§§§^
				Limonene (79)	None^++^
				**DPRA**	
				Anisyl alcohol (73)	None
				Pentachloropheneol (80)	SNAr (and/or possibly other mechanism)
				Hydrocortisone (85)	Schiff base/none*
				Phenoxyethanol (92)	None
				Salicylic acid (100)	None
				Phenol (104)	None
False negatives	22	19	11	**Common**	
				3-Dimethylaminopropylamine (10)	Schiff base^+^
				2-Methoxy-4-methylphenol (24)	Michael acceptor^+++,§§§^
				Coumarin (34)	Michael acceptor^§^
				Ethylene diamine (35)	Schiff base^+^
				Allyl phenoxyacetate (48)	SN2
				Benzyl Alcohol (52)	None
				Amylcinnamyl alcohol (54)	Michael acceptor/none*
				Aniline (55)	None^+^
				Geraniol (60)	Schiff base^+++^
				Hexyl salicylate (61)	None
				Resorcinol (67)	Michael acceptor^+,§§§§^
				**ProtReact**	
				Methylisothiazolinone (2)	SN2-reaction
				Diphencyclopropenone (6)	Acyl transfer
				Formaldehyde (11)	Schiff base
				Thioglycerol (21)	None/SN2*
				Glyceryl monothioglycolate (38)	None/SN2*
				2-Mercaptobenzothiazole (41)	Acyl transfer
				5-Methyl-2,3-hexanedione (42)	Schiff base
				Penicillin G (44)	Acyl transfer
				Benzocaine (56)	None
				Hydroxycitronellal (62)	Schiff base
				Methylmethacrylate (66)	Michael acceptor
				**DPRA**	
				Chlorpromazine (31)	Schiff base/none*^,+++^
				Farnesol (37)	Michael acceptor^++++^
				Cinnamyl nitrile (49)	Michael acceptor/none*
				Dibenzyl ether (51)	None
				Amyl cinnamic aldehyde (53)	Michael acceptor^§§^﻿
				Linalool (65)	None^++^
				β, β 3-Trimethyl benzenepropanol (69)	None
				Benzyl Cinnamate (70)	Michael acceptor***

^#^The numbers between the parentheses have a direct correspondence to the numeration in Table 1

^+^pro-hapten; ^++^pre-hapten; ^+++^pro/pre-hapten; and ^++++^pre/pro-hapten. Classifications retrieved from Hoffman et al. (2018)

Mechanistic domain of chemical reaction from Hoffman et al. (2018): *discordant results in Toxtree/OECD toolbox; ***Urbisch et al. (2015) and Toxtree also indicate SN2

^§^Definite assignment as a Michael acceptor; ^§§^Probably/possibly a Michael acceptor, but other possibilities cannot be ruled out; ^§§§^Definite assignment as a pro-Michael acceptor; ^§§§§^Probably/possibly a pro-Michael acceptor, but other possibilities cannot be ruled out. Classifications retrieved from Hoffman et al. (2018) and Roberts et al. (2007)

Of the 106 chemicals tested (71 sensitizers and 35 non-sensitizers), ProtReact correctly classified 79 compounds, while DPRA correctly classified 78 compounds. Of the correctly classified compounds, 65 were correctly classified by both assays/approaches (41 sensitizers and 24 non-sensitizers). From the 35 non-sensitizers, ProtReact classified 5 as sensitizers and DPRA classified 9 as sensitizers, of which 3 were concordant in both assays. Of the 71 sensitizers, ProtReact classified 22 as non-sensitizers and DPRA classified 19 as non-sensitizers, of which 11 were concordant in both assays (more than half, were pre/pro-haptens). DPRA false negatives included 3 pre/pro-haptens, which were correctly classified as sensitizers by the ProtReact assay (Table 4). The wide diversity of mechanistic domains of the misclassified chemicals hampers the association with a specific class of chemicals, although these results suggest that ProtReact has a better performance at classifying chemicals with no mechanistic domain alert, correctly classifying 28 out of 35, compared to DPRA, which correctly identified 23 out of 35.

## Discussion

The “Three Rs” principle has been present in European Union legislation since 1986 when the first EU law for animal protection for experimental and other scientific purposes was adopted. With the European Union ban on in vivo testing of cosmetics and toiletry ingredients, investigators have made an effort to develop NAMs for skin sensitization using in vitro, in silico, and in chemico approaches, which culminated in the development of several methods that are now fully developed and validated for skin sensitization hazard (Hoffmann et al. 2018, 2022). One of the first tests to be validated was DPRA (OECD TG 442C), which exhibited good predictivities when compared to local lymph-node assay data. DPRA has shown to be applicable to chemicals covering a wide variety of organic functional groups, mechanistic domains, sensitization potencies, and physicochemical properties; nevertheless, it also comprises some limitations (Seo et al. 2022). Indeed, the main disadvantage of DPRA is the requirement for specialized analytical instruments for the detection of residual peptides, such as HPLC with UV detection system and trained personnel to operate and validate the results. The process is time-consuming and expensive for evaluating many samples. Furthermore, poor solubility and co-elution of the test chemical with the peptides also represent limitations. To overcome these limitations, we have attempted to develop a new high-throughput screening assay to predict the sensitization potential of new chemicals based on a conventional spectrophotometric analysis. The developed assay here proposed—ProtReact is a simple, robust and cost-effective assay to rapidly identify skin sensitizers, and has shown to be as reliable as DPRA. ProtReact consists of two spectrophotometric assay methods based on a 96-well plate/test tube platform to assess the reactivity of chemicals to the amino (lysine) and thiol groups (glutathione) (Supplementary Figs. S1 and S2). Using ProtReact assay, we tested a dataset of 106 chemicals (71 sensitizers and 35 non-sensitizers) that were previously categorized by their sensitization potential according to human data (Basketter et al. 2014). The spectrophotometric assay for cysteine depletion was based on the method developed by Schultz et al. 2005 with some modifications. Given the characteristics of the spectrophotometric assay, data must be carefully interpreted for chemicals with unique color or that may interfere with spectrophotometric property changes upon reaction with peptides and/or reaction with the detection reagent. An alternative assay for these colored chemicals was herein performed with thiol-functionalized agarose beads, which allowed the removal of the unbound chemicals and, therefore, the removal of chemical color interference. The spectrophotometric assay using lysine peptide was similar to the spectrophotometric assay using cysteine peptide with some changes. Specifically, poly l-Lysine, FSE, as well as incubation with chemicals for 24 h were used instead of GSH, Ellman’s reagent, and incubation with chemicals for 1 h, respectively.

It is well known that a chemical able to react with any peptide to induce peptide depletion can be classified as a sensitizer. Therefore, we evaluated the prediction models for cysteine depletion and for the cysteine and lysine mean depletion. In ProtReact approach, chemicals were classified as sensitizers when cysteine depletion was equal or higher than 13.935% or the mean depletion equal or higher than 9.563% (Table 2). The 13.935% cut-off for cysteine depletion resulted in the best accuracy (75%), compared with mean-peptide depletion (73%), as well for other performance indicators, such as specificity and balanced accuracy (Table 3). We then compared the prediction value of the ProtReact assay with the prediction value of DPRA data (mean depletion). ProtReact 9.563% cut-off for mean-peptide depletion showed similar prediction values compared to DPRA assay (mean depletion), although ProtReact 13.935% cut-off for cysteine depletion showed higher accuracy, balanced accuracy, and specificity compared to the prediction value of DPRA data (mean depletion). The dataset of 106 chemicals comprised 35 chemicals lacking structure alerts, 33 Michael Acceptors (30 Michael Acceptors, 3 Michael Acceptor/none), 17 Schiff base (15 Schiff base and 2 Schiff base/none), 10 SN2 (8 SN2 and 2 none/SN2), 9 Acyl Transfers, and 2 SNAr, according to the Cosmetics Europe database (Hoffmann et al. 2018). Briefly, the mechanistic domains of chemical reactions were extracted in a sequential manner from several sources, although, when either the OECD QSAR toolbox or Toxtree predicted a domain and the other model did not, the predicted domain was used (Hoffmann et al. 2018). No clear correlation was found between chemical’s mechanistic domain and higher predictivity for both approaches. Nonetheless, DPRA showed better performances at classifying Acyl Transfers (correctly classifying 9 out of 9 Acyl Transfers sensitizers), while ProtReact only identified 6 and ProtReact showed better performances classifying chemicals without a structure alert (correctly classifying 28 out of 35, while DPRA only identified 23).

This is not the first attempt made by the scientific community to optimize DPRA. Indeed, two modified versions of DPRA, the amino acid derivative reactivity assay (ADRA) and kinetic DPRA (kDPRA), are already approved by OECD. ADRA is based on the same scientific principles of DPRA, although with different nucleophilic reagents [N-(2-(1-naphthyl)acetyl)-l-cysteine (NAC) and α-N-(2-(1-naphthyl)acetyl)-l-lysine (NAL)], which allowed testing of soluble chemicals at lower concentrations. Although ADRA is also based on HPLC–UV detection, since NAC and NAL have a naphthalene ring which has a known emission spectrum, Wanibuchi and colleagues developed an ADRA fluorescence detection method (ADRA-FL), which showed similar results to ADRA-UV for a set of 82 chemicals (accuracies of 88% and 87%, respectively) (Wanibuchi et al. 2019). However, ADRA-UV and ADRA-FL rely on the use of HPLC, which is not always available in most labs and is often expensive and time-consuming, requiring expert personnel. Unlike DPRA and ADRA, kDPRA only measures reactivity with the cysteine peptide and does not rely on HPLC–UV equipment, but rather on a fluorescence plate reader, although cysteine peptide reactivity is evaluated at six time-points (10, 30, 90, 150, 210, and 1440 min) and at five concentrations (5, 2.5, 1.25, 0.625, and 0.3125 mM). Recently, Natsch evaluated the predictivity of kDPRA based on human data of 123 chemicals and obtained a balanced accuracy of 76%, sensitivity of 64% (21/33), and a specificity of 89% for predicting GHS 1A sensitizers (Natsch et al. 2020). In 2014, Cho and colleagues also proposed a new spectrophotometric assay method (Spectro-DPRA), performed in 96-well plates, to determine the reactivity of chemicals toward two chemical groups, the thiol group of a cysteine-containing peptide (cysteine peptide) detected using DTNB and the amino group of a lysine-containing peptide (lysine peptide) detected using the amine reactive dye fluorescamine. Chemicals were classified as sensitizers when they induced more than 10% depletion of cysteine peptides or more than 30% depletion of lysine peptides. The authors reported a sensitivity, specificity, and accuracy of 80.0%, 86.7%, and 82.5%, respectively, for a dataset of 40 chemicals (25 sensitizers and 15 non-sensitizers) (Cho et al. 2014). In 2022, a pre-validation study for Spectro-DPRA was published, and for a dataset of 54 substances (33 sensitizers and 21 non-sensitizers), the authors reported a sensitivity, specificity, and accuracy values of 87.9%, 90.5%, and 88.9%, respectively (Seo et al. 2022). In both studies, the predictive rates of Spectro-DPRA were based on LLNA. Furthermore, the dataset used was considerable smaller than our dataset. As reported by Hoffman and colleagues, the predictivity of the LLNA compared to human data is only about 74.2%, therefore ADRA and Spectra-DPRA performances must be interpreted carefully. Hoffman and colleagues also reported predictivity capacities of 71.4% to 74.2% for DPRA, using human data (with sample sizes of more than 100 chemicals) (Hoffmann et al. 2018, 2022), which is in line with our results both for ProtReact and DPRA. Compared to DPRA, ADRA, and Spectra-DPRA, ProtReact cysteine depletion assay strongly decreases the incubation period from 24 to 1 h. Furthermore, ProtReact lysine depletion assay uses about one-hundredth of the amine reaction probe compared to Spectra-DPRA (10 µM of FSE and 1 mM fluorescamine, respectively). Relatively to kDPRA, ProtReact presents similar results (balanced accuracies of 76% and 72%, respectively) with just one concentration of test chemicals (5 mM) and one time point (1 h), instead of five concentrations and six time-points. Last but not least, no direct extrapolations can be made between the different assays presented (ADRA, kDPRA, and Spectra-DPRA), because their prediction performances come from different datasets. For a correct comparison between assays, a unified dataset of chemicals must be first defined and then applied. Nevertheless, the accuracies/balanced accuracies were very similar between all the assays presented.

Taking together, the results herein presented show that ProtReact assay can be used as an alternative assay for identifying skin sensitizers, with several advantages, also maintaining similar performances to the already OECD approved test methods. ProtReact assay advantages include: (i) the use of a fluorescent plate reader (high-throughput) instead of HPLC–UV equipment; (ii) a predicate capacity using only one type of peptide depletion (cysteine) at short incubation time period; and (iii) a fixed concentration of test chemical (simplicity). Furthermore, and for the best of our knowledge, our cysteine depletion assay with thiol-functionalized agarose beads is the only assay that allows washing of unbound chemicals, before the addition of the thiol-reactive dye, therefore minimizing the interference of the test chemical with the dye.

In summary, we established two spectrophotometric methods by determining the reactivity of chemicals to the thiol group of GSH and to the amino group of a lysine peptide using Ellman’s reagent and FSE as detecting reagents of free thiol and amine groups, respectively. We then examined the possibility of using them as in chemico sensitization test methods by testing 106 chemicals that were previously well categorized by their human sensitization potential. The most promising prediction model, with an accuracy of 75%, and specific of 86% resulted from the 13.935% cut-off for cysteine depletion. Although this method has some limitations and needs further improvement and optimization to be included as an official risk and hazard assessment method, these results demonstrate that spectrophotometric methods could serve as easy, fast, and high-throughput screening tools for the prediction of skin sensitization potential of haptens. Additionally, it can be easily performed in any lab (uses only a spectrophotometer) and ProtReact cysteine depletion assay is less time-consuming (1.5 h versus 24 h for DPRA). Although ProtReact and DPRA show some discrepancies in terms of concordant classifications, their predictive performances, namely accuracy, are quite similar. Therefore, and since no single in vitro skin sensitization assay is approved as a stand-alone method for risk assessment, ProtReact could be part of a Defined Approach or IATA, like the DPRA. Accordingly, it would be interesting to explore this approach for regulatory purposes. Furthermore, ProtReact cysteine depletion assay may be transposable for 96-well plates, further improving the assay throughput.

## Supplementary Information

Below is the link to the electronic supplementary material.

Supplementary file 1 (DOCX 17605 KB)

Supplementary file 2 (XLSX 45 KB)
