# Supplementary material for: Development of an in chemico high-throughput screening method for the identification of skin sensitization potential
Source: Arch Toxicol. 2023 Jul 19;97(9):2441–51. doi: 10.1007/s00204-023-03550-z (PMC10404171; doi:10.1007/s00204-023-03550-z)
Supplement: Supplementary file 1 — (DOCX 17605 KB) [file 204_2023_3550_MOESM1_ESM.docx]

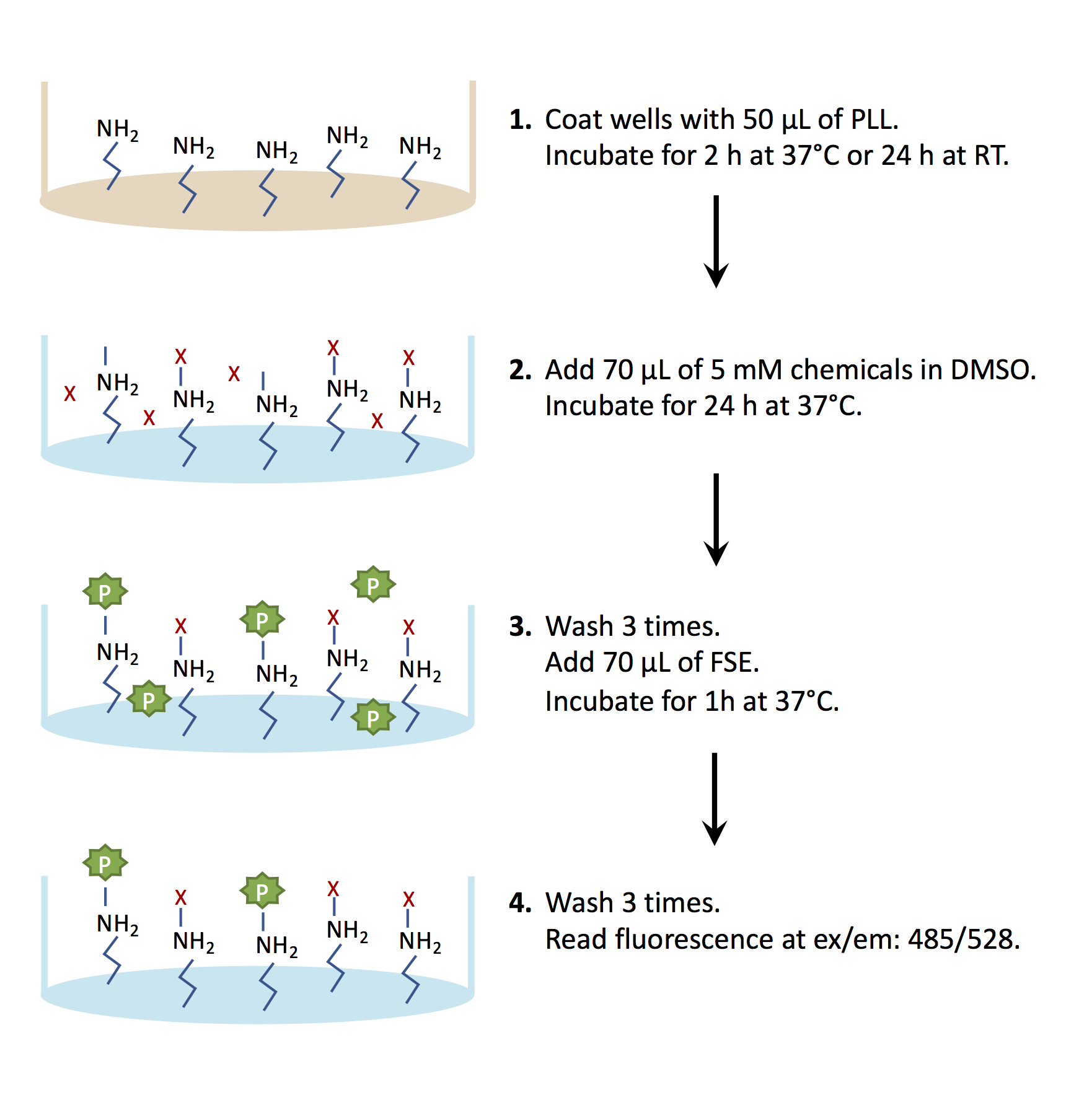


**Supplementary Fig. S1** Schematic representation of ProtReact Lysine depletion Assay. PLL - Poly-L-Lysine, FSE - Fluorescein-5-EX, Succinimidyl Ester

**
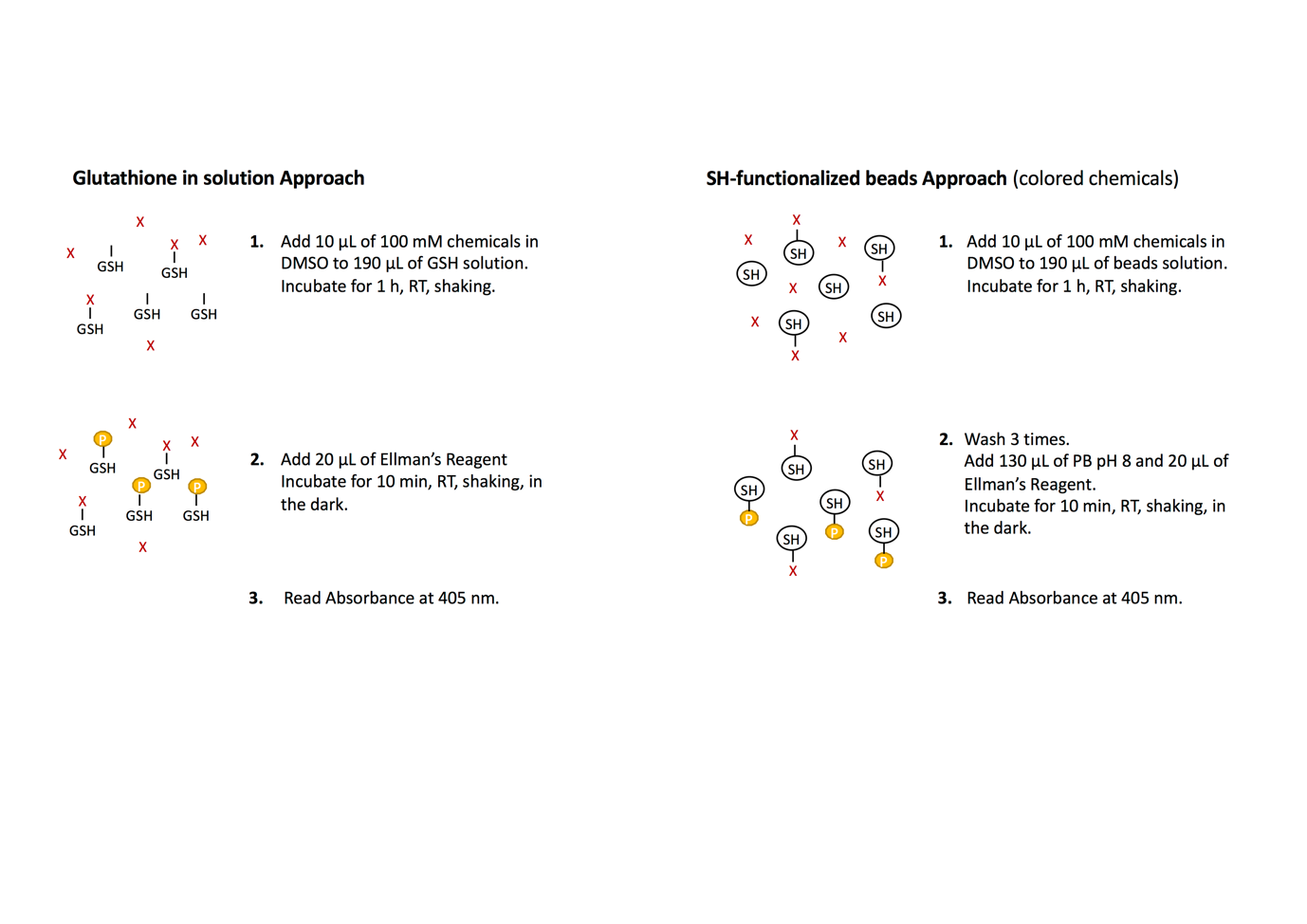
**

**Supplementary Fig. S2** Schematic representation of ProtReact Cysteine depletion Assay. GSH – Glutathione, PB – Phosphate Buffer, RT – Room Temperature

**Supplementary Fig. S3** Lysine depletion comparison between ProtReact and DPRA assays. The vertical doted lines correspond to the human potency (P) categorization according to Basketter *et al*., 2014. DPRA data retrieved from Hoffmann *et al*., 2018. Sensitizers: P1-P4; Non-sensitizers: P5 and P6. Lys, lysine

**Supplementary Fig. S4** Cysteine depletion comparison between ProtReact and DPRA assays. The vertical doted lines correspond to the human potency (P) categorization according to Basketter *et al*., 2014. DPRA data retrieved from Hoffmann *et al*., 2018. Sensitizers: P1-P4; Non-sensitizers: P5 and P6. Cys, cysteine
